# Serum biochemistry in cows of different breeds presented with reproductive conditions

**DOI:** 10.4102/ojvr.v86i1.1742

**Published:** 2019-08-29

**Authors:** Keitiretse Molefe, Mulunda Mwanza

**Affiliations:** 1 Department of Animal Health, Faculty of Natural and Agricultural Science, North-West University, Mafikeng, South Africa

**Keywords:** animal health, cow, metabolic disorders, nutrition, reproductive performance, serum biochemistry

## Abstract

Minimising health problems and increasing yield have always been the objectives in livestock agriculture. Hence, increases in incidences of reproductive conditions in cattle farming pose a great threat to productivity and impose undesirable economic implications. This study aimed to examine the concentrations of different biochemical compounds in cows with reproductive conditions. Seventy-seven blood samples were collected from cows at different rural areas around Mafikeng, following cases of downer cow syndrome, dystocia, retained placenta, vaginal prolapse and abortion. Means of serum metabolites across the different reproductive conditions were statistically compared using Pearson’s chi-square test to determine variations of serum metabolites in cows of different breeds. In mixed breed cows, higher than normal calcium concentrations were observed in downer cow syndrome (25.25 ± 8.47) and dystocia (85.50 ± 8.46) cases. It was also observed that cholesterol concentrations were significantly low in abortion (2.52 ± 0.79), retained placenta (3.18 ± 0.61) and vaginal prolapse (2.37 ± 0.97) cases in Afrikaner cows. The study showed that Brahman (43.1%) and Afrikaner (43.1%) breeds were mostly affected by downer cow syndrome. Additionally, the occurrences of downer cow syndrome (53.9%) and abortions (60%) were mostly observed in cows of 1–3 years, in second and first parities, respectively. This study proves that concentrations of calcium, urea or blood urea nitrogen (BUN), magnesium and cholesterol are significantly altered in incidences of reproductive conditions in cows of different breeds. It is also shown that serum biochemistry is affected by reproductive conditions in cows of different ages and parity. This data serves as a tool that could be used to enhance research in animal production and reproduction.

## Introduction

The use of serum metabolic profiling in veterinary medicine is a significant test in herd health assessment (Oetzel 2004). The physiological and nutritional status of cows can be evaluated using the analysis of blood biochemical parameters (Ashmawy 2015). Irregularities in numerous biochemical factors have been held liable for reproductive failures in cows (Bazzano et al. 2016). The cow’s immune capacity during the transition period greatly influences its susceptibility to reproductive and metabolic disorders (Azawi 2008). The incidences of reproductive conditions are normally elevated during the transition period (Santos, Rutigliano & Filho 2009).

Disturbances in the normal function of reproductive processes leading to dystocia can severely affect cattle production (Savc, Kenny & Beltman 2016). Associated risk factors and causes of dystocia include direct factors (e.g. uterine torsion and foetal mal-presentations), phenotypic factors (calf birth weight, multiple calving, prenatal mortality, cow pelvic area, cow body weight, body condition at calving and gestation length), non-genetic and genetic factors (Gaafar, El-Lateif & El-Hady 2011; Mee 2012; Zaborski et al. 2009). Dystocia is an undesirable condition because of its impact on cows’ subsequent reproductive performance and negative economic effects on farm production. The estimated cost of dystocia in any parity on average is approximately $24.24, which is approximately R323.78 (South African rand) as per case attendance (Dematawewa & Berger 1997).

Vaginal prolapse is another important reproductive health problem seen in cows. It is a condition of the reproductive tract frequently seen in 24-month-old cows, either pregnant or non-pregnant (Do Nascimento, Mello & Corrêa 2016). A study of Abuom et al. (2012) revealed that the incidences of vaginal prolapse increased during the dry season as a consequence of poor nutrition leading to loss of body condition as well as the peri-vaginal fat, which is important in supporting the uterus and vagina within the pelvic cavity. Also, foods containing oestrogenic substances such as subterranean clover pasture, soya-bean meal, mouldy maize and barley may result in a high incidence of vaginal prolapse (Noakes, Parkinson & England 2001).

Bovine abortions are complex reproductive conditions that occur between 42 and 280 days of pregnancy which may result from several pathogenic agents such as bacterial, viral, fungal, protozoal and non-infectious agents (Anka et al. 2014). The cost of production losses due to abortions is estimated to be about $640.00 (or R8548.64) per abortion case (Gädicke, Vidal & Monti 2010). Implementation of precautionary measures such as good health management, sufficient nutrition and other environmental factors affecting reproduction during pregnancy are important aspects to assure reduction in the high cost of animal losses through abortions.

The deficiency in secretions of oxytocin, prostaglandins (PGF_2__α_) and serum calcium levels, dystocia and delay in the involution of the uterus may cause retained placenta (Akar & Yildiz 2005). Premature induction of parturition with glucocorticoids and/or prostaglandins, low plasma oestrogen concentration, deficiency in vitamin E and selenium, negative energy balance, hypocalcaemia hormonal disorders and others factors predispose cows to the incidences of retained placenta (Jemal 2016).

Downer cow syndrome is defined as lateral or sternal recumbency that persists longer than 24 hours whereby the animal is unable to rise to a standing position (Ménard & Thompson 2007:487). The aetiology of recumbency of the animal is not well understood; however, the association of energetic or electrolyte metabolism as well as infectious diseases and trauma may result in this syndrome (Guyot et al. 2017). The main causes of downer cow syndrome are hypocalcaemia and energy imbalance (Radostits et al. 2000). Other factors related to the incidences of downer cow syndrome include post-parturient disorders such as fatty liver, ketosis, metritis, nerve paralysis and mastitis (Kimura, Reinhardt & Goff 2006).

The association between nutrition, diseases and reproductive performance has been a subject of increasing interest to producers, veterinarians and nutritional researchers in recent years (Martinez et al. 2014; Pradhan & Nakagoshi 2008). The multifactorial nature of the reproductive conditions seriously limits the effectiveness of treatment methods and increases the risk of misdiagnoses. Note that it is difficult to monitor the level of impact reproductive conditions have on cattle production, mainly because of lack of data from diagnostics (Thornton 2010). Consequently, cattle production efficiency remains in a critically hindered status, as farmers still face challenges in their attempt to reduce reproductive health illnesses. Therefore it is necessary to conduct this study in order to bring about a guiding tool of variations in serum metabolites in cows of different breeds presenting with downer cow syndrome, dystocia, retained placenta, vaginal prolapse and abortion.

## Materials and methods

### Study design

The study was conducted in the Mafikeng area, found in the North-West Province in South Africa. The geographical coordinates of the study area are 25°51′S and 25°38′E. Blood samples were collected from communal farms around Mafikeng. Samples were collected when cases of reproductive conditions were reported to the North-West University Dale Beighle Centre for Animal Health Studies.

A total of 77 blood samples were collected from cows with cases of downer cow syndrome (*n* = 13), dystocia (*n* = 14), retained placenta (*n* = 13), vaginal prolapse (*n* = 9), and abortion (*n* = 28). Blood was collected from the jugular vein using an 18G double-pointed vacutainer needle into clot activator red stopped tubes (Greiner Bio-One, Kremsmunster, Austria; Germany). Samples were placed on ice and transported to the North-West University animal health laboratory for analysis. Serum was obtained by centrifugation (2500 revolutions per minute for 10 minutes) immediately after collection and was stored at -20 °C until assay. The IDEXX Catalyst Dx^®^ Chemistry Analyzer was used for blood biochemistry testing of serum metabolites to determine concentrations of calcium, magnesium, total bilirubin, cholesterol (CHOL), ammonia, triglycerides, urea, uric acid and aspartate amino-transferase (AST) following the procedure of the manufacturer (IDEXX Laboratories Inc 2014, Westbrook, ME, United States [US]). Information about the age, parity and breed of the cows were also recorded.

Descriptive statistics (frequencies and percentages) were used to determine ages and parities of cows affected by reproductive conditions. The measure of association was performed using Pearson’s chi-square test to determine association between cow breeds (Afrikaner, Brahman, Charolaise, Mixed, Nguni, Drakensberger and Bonsmara) and blood chemistry (calcium, magnesium, total protein, creatinine kinase, lipase, triglycerides, urea or BUN, uric acid, AST, sodium, chloride, potassium, CHOL, total bilirubin and ammonia) in the different groups (cows experiencing downer cow syndrome, dystocia, retained placenta, vaginal prolapse and abortion) and different concentrations of serum metabolites, and the interaction (reproductive condition × cow breed) is indicated in the results. Data were analysed statistically in Statistical Analysis Software (version 20) using the analysis of variances techniques (ANOVA). The results for serum metabolites concentrations were expressed as means ± SE. Significance levels for all the tests were set at *p* < 0.05.

### Ethical considerations

The study was approved by the Animal Ethics Committee at the North-West University (NWU-00409-18-S5).

## Results

This study aimed to examine the concentrations of different biochemical compounds in different breeds of cows with reproductive conditions. The data in this section also shows the distribution of affected cows between ages and parities of affected cows.

This study showed significant (*p* < 0.05) variations regarding the reproductive condition × Cow breed interactions seen in concentrations of urea or BUN, calcium, AST, total bilirubin, lipase, potassium and total protein (Table 1). Additionally, the serum concentrations of triglyceride, creatinine kinase, sodium, chloride and total protein did not show a significant difference in association to the conditions (*p* > 0.05), as indicated in Table 1.

**TABLE 1:** *p*-Values obtained after statistical analysis of variance between average serum metabolites concentrations in cows affected by reproductive condition without the breed distinctions and within different breeds.

Parameter	Reproductive condition (1)	Reproductive condition × Cow breed (2)
Urea or BUN	0.01149*	0.0051**
Uric acid	0.3356	0.8118
Calcium (Ca)	0.2808	0.0347*
Magnesium (Mg)	0.0001**	0.5977
AST	< 0.0001**	0.0002**
TBIL	< 0.0001**	< 0.0001**
Cholesterol	0.2912	0.0931
Triglycerides	0.8005	0.7535
Ammonia	0.8709	0.6451
Lipase	0.0106*	0.0357*
Creatinine kinase (ck)	0.9659	0.6099
Sodium (Na)	0.9624	0.9654
Potassium	0.0184*	< 0.0001*
Chloride (Cl)	0.1918	0.9921
Total protein (Tp)	0.2451	0.0465*

Reproductive condition (1): Without breed interactions between levels of serum metabolites in reproductive condition; Reproductive Condition × Cow breed (2): Within interactions between serum metabolite concentrations from different reproductive conditions.

TBIL, total bilirubin; BUN, blood urea nitrogen.

*, ** , Show significant differences in each row (*p* < 0.05; *p* < 0.001), respectively.

The level of significance within a particular reproductive condition and breed is further elaborated in subsequent tables. In this section the tables indicate the mean variations of different serum metabolites in cows presented with reproductive conditions within a specific breed showing significant differences.

Table 2 shows significantly high serum mean concentrations of calcium in mixed breed cows which experienced downer cow syndrome (25.25 ± 8.47 mmol/L) and dystocia (85.50 ± 8.46 mmol/L). Significant difference in calcium concentrations was not observed in other reproductive conditions.

**TABLE 2:** Mean serum calcium (Ca) concentrations in different cow breeds experiencing reproductive conditions.

Condition	Breed	Ca (Mean ± SE)	*p*-Value
Downer cow syndrome	Afrikaner	2.07 ± 9.76	0.8324
	Brahman	1.98 ± 9.76	0.8393
	Charolaise	0.78 ± 11.95	0.9482
	Mixed	25.25 ± 8.47*	0.0038
Dystocia	Afrikaner	2.14 ±7.56	0.7776
	Brahman	2.42 ± 11.96	0.8402
	Mixed	85.50 ± 8.46**	< 0.0001
	Nguni	2.12 ± 11.95	0.8598

Ca, calcium.

*, ** , Means with superscripts differ significantly (*p* < 0.001); calcium normal range (2.0–2.8 mmol/L) was extracted from Kahn, Line and Station (2010).

The Table shows higher mean concentrations of urea in aborting Bonsmara cows (18.5 ± 3.04 mmol/L), Charolaise cows with downer cow syndrome (10.35 ± 3.04 mmol/L) and Afrikaner cows with vaginal prolapses (14.7 ± 2.15 mmol/L), while low mean urea (1.45 ± 3.04 mmol/L) concentrations were seen in Brahman cows with dystocia (Table 4).

**TABLE 3:** Mean serum magnesium concentrations in cows presenting abortion, downer cow syndrome and dystocia in different breeds.

Condition	Breed	Mg (Mean ± SE)	*p*
Abortion	Afrikaner	0.57 ± 0.19*	0.0033
	Bonsmara	0.61 ± 0.32	0.1231
	Brahman	0.67 ± 0.13	0.1000
	Drakensberger	0.67 ± 0.26	0.1518
	Nguni	0.84 ± 0.23	0.1250
Downer cow syndrome	Charolaise	0.46 ± 0.33	0.1637
	Mixed	1.33 ± 0.23**	< 0.0001
Dystocia	Mixed	2.12 ± 0.23***	< 0.0001

Note: Low mean concentrations of magnesium were observed in aborting (0.57 ± 0.19 mmol/L) Afrikaner cows (*p* < 0.05), as shown in Table 3. In addition, high mean concentrations of magnesium in mixed breed cows were detected in downer cow syndrome (1.33 ± 0.23 mmol/L) and dystocia (2.12 ± 0.23 mmol/L) cases, as indicated in Table 3.

Mg, magnesium.

*,**,*** , Means with superscripts in each row are significantly different (*p* < 0.05). Magnesium reference range (0.6–1.2 mmol/L) was extracted from Ode, Adamu & Saror (2017).

**TABLE 4:** Mean serum urea or blood urea nitrogen concentration in different cow breeds experiencing reproductive conditions.

Condition	Breed					
	Brahman	Bonsmara	Nguni	Afrikaner	Drakensberger	Charolaise
Abortion	3.57 ± 1.24	18.5 ± 3.24**	2.70 ± 2.15	5.50 ± 3.14	3.07 ± 2.48	4.20 ± 3.11
Retained placenta	4.20 ± 2.48	2.70 ± 1.48	3.00 ± 1.22	4.32 ± 1.36	5.52 ± 2.24	3.40 ± 1.28
Downer syndrome	4.40 ± 1.18	4.40 ± 2.08	4.31 ± 3.14	4.90 ± 2.48	3.40 ± 1.18	10.35 ± 3.04****
Dystocia	1.45 ± 3.04*	3.40 ± 2.40	5.85 ± 3.02	4.94 ± 1.92	5.85 ± 3.04	5.01 ± 3.04
Vaginal prolapses	3.10 ± 2.03	4.00 ± 2.28	3.98 ± 1.92	14.7 ± 2.15***	3.45 ± 1.18	4.40 ± 2.18

*,**,***,**** , Values within a row with different superscripts differ significantly at *p* < 0.05. Urea reference range (2.5–6.1 mmol/L) was extracted from IDEXX laboratory instruction manual of 2014.

In aborting cows, CHOL mean concentrations were low in Afrikaner, Bonsmara, Brahman, Drakensberger and Nguni cows (Table 5). Moreover, cows presenting with downer cow syndrome showed low CHOL concentrations in Afrikaner, Brahman and mixed breed cows, as seen in Table 5. Low CHOL mean concentrations were also noted in retained placenta and vaginal prolapse in Afrikaner cows, as indicated in Table 5. Other findings revealed that Brahman, Afrikaner, mixed breed and Nguni cows with dystocia had significantly low CHOL mean concentrations, as shown in Table 5.

**TABLE 5:** Mean serum cholesterol concentrations in cows experiencing reproductive conditions in different breeds.

Condition	Breed	Cholesterol mean ± SE	*p*
Abortion	Afrikaner	2.52 ± 0.79**	0.0021
	Bonsmara	2.95 ± 1.37*	0.0347
	Brahman	3.53 ± 0.56**	< 0.0001
	Drakensberger	2.67 ± 1.12*	0.0195
	Nguni	3.19 ± 0.97**	0.0015
Downer cow syndrome	Afrikaner	2.83 ± 1.12*	0.0135
	Brahman	2.17 ± 1.12*	0.0564
	Charolaise	1.90 ± 1.37	0.1702
	Mixed	6.50 ± 0.97**	< 0.0001
Dystocia	Afrikaner	3.06 ± 0.87**	0.0007
	Brahman	3.68 ± 1.37**	0.0089
	Mixed	7.75 ± 0.97**	< 0.0001
	Nguni	4.04 ± 1.37**	0.0043
Retained placenta	Afrikaner	3.18 ± 0.61**	< 0.0001
Vaginal prolapses	Afrikaner	2.37 ± 0.97**	0.0170

SE, standard error.

*,** , Means with superscripts in each row are significantly different (*p* < 0.05). Cholesterol normal range (71–156 mmol/L) extracted from IDEXX laboratory instruction manual of 2014.

**FIGURE 1:**
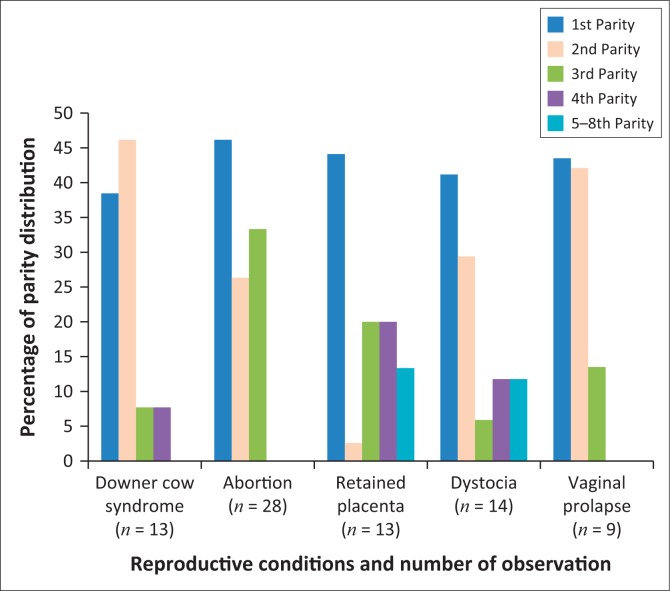
Parity distribution of cows affected by reproductive conditions parities.

The above graphical representation indicated abortion (46.15%), retained placenta (44.1%), dystocia (41.18%) and vaginal prolapse (43.5%) cases of cows in the first parities (Figure 1), whereas downer cow syndrome (46.15%) and vaginal prolapses (42.10%) mostly occurred in the second parity cows (Figure 1).

**FIGURE 2:**
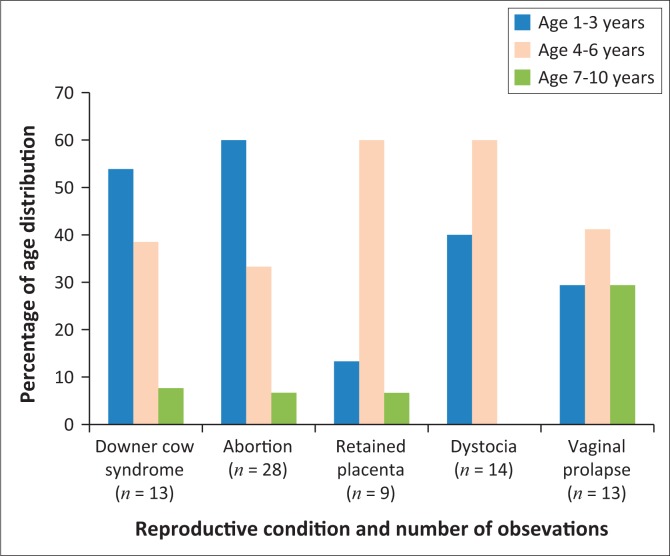
Age association with the occurrence of reproductive conditions in cows.

The histogram above shows that approximately 53.9% of downer cow syndrome and 60% of abortion cases were seen in cows of ages 3–4 years (Figure 2). Amongst other conditions, retained placenta (60%), dystocia (60%) and vaginal prolapses (41.2%) occurred in cows of ages 5–6 years. Additionally, the frequencies in 7–10 years old cows were very minimal showing low distribution amongst the occurring reproductive conditions (Figure 2).

## Discussion

Poor reproductive capacity in cow herds has negative implications on farm profitability as financial losses occur through high treatment and replacement costs, which consequently decreases farm income aptitudes (Gädicke et al. 2010). Constant herd health assessment can help reduce the impact of reproductive losses by minimising the costs of veterinary services. This study aimed to determine differences in the mean concentrations of serum metabolites in cows affected by reproductive conditions. In the current study, concentrations of calcium, urea or BUN, AST, total protein, total bilirubin, potassium and lipase significantly (*p* ≤ 0.05) varied in the breed and reproductive condition interactions (Table 1). Additionally, no significant differences were seen in concentrations of triglyceride, creatinine kinase, sodium and chloride and total protein did not show significant difference in relation to the incidences of reproductive conditions (*p* > 0.05).

In our study the mean concentrations of calcium indicated significantly high concentrations in cases of both downer cow syndrome (25.25 ± 8.47 mmol/L) and dystocia (85.50 ± 8.46 mmol/L) in mixed breed cows. On the contrary, the incidence of downer cow syndrome has been linked with low calcium levels (Benzaquen et al. 2015:186). However, such associations were not seen in this study, because the concentrations of calcium in most of the cows were within the normal ranges (2.0–2.8 mmol/L).

Similar observations were reported by McDougall (2001:60–67), where no relationship was established between low blood calcium, reproductive performance and dystocia. High levels of calcium in downer cow syndrome may be interpreted as a result of hormonal imbalance, for instance excessive secretion of parathyroid hormone (PTH) causing excessive bone resorption (Balamurugan et al. 2017:694), which could explain the increased calcium level in downer cows observed in this study. In downer cow syndrome, low mean concentration of calcium was observed in Brahman (1.98 ± 9.76 mmol/L) and Charolaise (0.78 ± 11.95 mmol/L) breed cows; however, no significant differences were indicated in the current study. This may be because of a lack of homogeneity in the number of cows within the different breeds, because low calcium is a known indicator for downer cow syndrome (Benzaquen et al. 2015:186).

A significantly different magnesium concentration below normal was seen in aborting Afrikaner cows in this experiment. Similar observations were reported by Musa, Lanyasunya & Mukisira (2016:117), indicating a magnesium deficiency in aborting cows. The results also indicated magnesium concentrations with no significant differences (*p >* 0.05) in aborting cows of Brahman and Nguni breeds. Magnesium mean concentrations in downer cow syndrome and dystocia were significantly high in mixed breed cows. These results agree with those recently reported by Adams, Ishler & Moore (2017:96), indicating that a rise in blood magnesium levels is usually observed in downer cow syndrome because of the decrease in calcium levels. Moreover, sufficient calcium levels are required to prevent dystocia and downer cow syndrome, because of its functional characteristics in muscle contraction and relation (Goff 2008:45).

The present study also showed significantly higher urea mean concentrations in aborting (18.5 ± 3.04 mmol/L) Bonsmara cows and downer cow syndrome (10.35 ± 3.04 mmol/L) Charolaise cows. Our findings agree with previous works reported, showing that high urea concentrations may lead to abortions in cows (Rhoads et al. 2009:1986). Other observed results indicated low urea levels in dystocia (1.45 ± 3.04 mmol/L) Bonsmara cows. Cholesterol mean concentrations of aborting cows were significantly low in different breeds experimented in the present study. Negative energy balance, poor body condition and low CHOL concentrations are usually indicators for postpartum health illnesses and poor nutrition status in cows (Sepúlveda-Varas et al. 2015:e0122317), which could explain the low level of CHOL in aborting cows.

In downer cow syndrome cows, significantly low CHOL concentrations were noted in Afrikaner (2.83 ± 1.12 mmol/L), Brahman (2.17 ± 1.12 mmol/L) and mixed breed cows (6.50 ± 0.97 mmol/L), as seen in Table 5. Civelek et al. (2011:341) reported similar results. These results imply that when calcium levels are decreased, the concentration of CHOL is likely to drop. Significantly low CHOL mean concentrations were also noted in retained placenta (3.18 ± 0.61 mmol/L) and vaginal prolapse (2.37 ± 0.97 mmol/L) in Afrikaner cows, as indicated in Table 5. The current results are in agreement with the study of Civelek et al. (2011:341) which reported lowered CHOL level in cows-retained placenta.

The results of this study showed that the incidences of dystocia were most frequent in cows of ages 5–6 years and in the first parity (Figures 1 and 2). Similar data indicating a direct relationship between dystocia, age and parity (more frequent in primiparous than high parity cows) have previously been presented (Abera 2017:1–9; McDougall 2001:60–67). The occurrence of dystocia have dire economic implications on production, as cows become more susceptible to post-partum illnesses, most likely to die soon after parturition and encounter increased calve mortalities (De Amicis et al. 2018:104).

According to Mee (2008:933–101), the cases of downer cow syndrome are mostly seen in older cows. However, in this study, the cases of downer cow syndrome were more prominent in cows of ages 3–4 than in those of 7–10 years (Figure 2). Similar results have been described, showing that age has an impact on the incidence of metabolic disorders as the level of susceptibility may vary according to age, and moreover, heifers are more susceptible to downer cow syndrome (Kutanaee et al. 2014:367).

The cases of retained placenta were observed (60%) in cows of ages 5–6 years in their first (44.1%) parity (Figures 1 and 2). The results agree with those of Sharma et al. (2017:3103) which indicated (40.22%) incidence in primiparous cows. Previous studies have also stated that incidences of retained placenta in most cases increase because of downer cow syndrome and dystocia (McDougall 2001:60–67). This study revealed that vaginal prolapses were most frequent in cows of ages 5–6 years in the first parity (44.1%), as seen in Figures 1 and 2. Similarly, prolapse of the vagina has been reported to be influenced by cow age and parity (Sarma, Das & Nath 2017:1067). These results therefore suggest that metabolic profile testing is a good indicator for bovine reproductive conditions.

## Conclusion

The differences in serum metabolites concentrations were seen in cows of different breeds with reproductive conditions. Particularly, variations of serum calcium, magnesium, urea or BUN, AST and CHOL concentrations was observed in cows affected by dystocia, retained placenta, downer cow syndrome, vaginal prolapse and abortion. This study also showed that Nguni and Bonsmara breeds were the least affected by the reproductive conditions, suggesting that they are the most ideal to use in natural pasture rearing of cows. It is also indicated that age and parity could influence the change in blood chemistry as the cases of reproductive conditions were noted in cows of varying age groups and parities. The information obtained in this study serves as a useful tool for farmers as it can help them make informed decisions in determining the best animals to use for production.
